# Methylation-specific qPCR for the EBV C promoter to quantify EBV methylation

**DOI:** 10.1186/s13027-025-00702-x

**Published:** 2025-10-30

**Authors:** Logan George, Paul G. Rubinstein, Jennifer Petr, Ariela Noy, Lisa Haley, Emily Adams, Rena R. Xian, Richard F. Ambinder

**Affiliations:** 1 Department of Oncology, Johns Hopkins School of Medicine, 1650 Orleans Street, CRB1 Rm 389, Baltimore, MD 21287 USA; 2 Section of Hematology/Oncology, Department of Medicine, University of Illinois, Chicago, IL USA; 3 Section of Hematology/Oncology, Department of Medicine, Cook County Health and Hospital Systems (Cook County Hospital), Chicago, IL USA; 4 Weill Cornell Medical College, Cornell University, New York, NY USA; 5 Memorial Sloan-Kettering Cancer Center and Weill Cornell Medical College, New York, NY USA; 6 Department of Pathology, Johns Hopkins School of Medicine, Baltimore, MD USA

**Keywords:** DNA methylation, Epstein-Barr virus, Bisulfite, Hodgkin lymphoma, Plasma DNA

## Abstract

**Background/Objectives:**

Epstein-Barr Virus (EBV) is a ubiquitous virus associated with a variety of diseases including cancers. Evidence has emerged that the C promoter is methylated in many EBV-associated malignancies, whereas in free virion DNA it is unmethylated. We have developed and evaluated a methylation-specific PCR assay for the EBV C Promoter (MSPCP) that can be applied to human biological specimens to quantify EBV methylation.

**Methods:**

Two sets of methylation-specific primers were designed to anneal to bisulfite-converted DNA sequences with 3 CpGs in the forward primer binding site, and 2 CpGs in the reverse primer binding site. We evaluated this method in synthetic oligonucleotides, DNA extracted from cell lines, virion supernatants, and a variety of clinical specimens. EBV methylation of Cp, as measured by MSPCP, was validated with two orthogonal methods in select samples.

**Results:**

In contrived samples, this method had a linear range between 0–100% methylation. Application of this assay to DNA extracted from 11 formalin-fixed paraffin-embedded biopsy specimens showed high-level C promoter methylation in EBV-associated tumors (94–100%) but not in EBV-associated lymphoid hyperplasia. High-level EBV methylation was also detected in cell-free DNA extracted from the plasma of 13 patients with EBV-associated Hodgkin lymphoma. In contrast, EBV methylation was either not-detected, or detected at very low levels, in saliva from 25 adults in a general university population consistent with the presence of virion DNA.

**Conclusions:**

MSPCP is a simple, rapid and accurate method that characterizes the methylation status of the EBV C promoter, which may be useful in a variety of research and clinical settings.

**Supplementary Information:**

The online version contains supplementary material available at 10.1186/s13027-025-00702-x.

## Background

There is growing interest in the characterization of CpG methylation of cell-free DNA (cfDNA) in plasma for the detection and monitoring of cancers [1]. For example, multicancer early detection approaches have demonstrated that cfDNA methylation profiling of the human genome can detect a variety of cancer types [2]. Many groups have investigated targeted approaches to detecting DNA methylation associated with cancer [3–7]. A substantial fraction of human cancers is associated with viral pathogens. Epstein-Barr virus (EBV) is associated with Burkitt lymphoma, Hodgkin lymphoma, nasal NK/T cell lymphoma, gastric carcinoma, nasopharyngeal carcinoma (NPC) and post-transplant lymphoproliferative disease [8], among others. Detection of EBV DNA in plasma is sensitive in identifying individuals with EBV-associated cancers, but the results are not entirely specific [9–11]. The problem with EBV DNA as a tumor marker is its ubiquity. More than 90% of the world’s adult population is infected and viral DNA is readily detected in saliva and lymphocytes from healthy individuals [12, 13]. In higher risk groups, such as people living with HIV, EBV DNA is even more frequently detected in body fluids [14, 15].

The EBV life cycle is characterized by two distinct phases: latency and lytic replication [12]. In latency, there is restricted gene expression and no production of virions. In lytic replication, there is broad expression of viral genes with virion production. Transmission of EBV predominantly occurs through saliva [13]. Virion DNA lacks methylation, histones and other epigenetic marks [16], whereas episomal viral DNA acquires these epigenetic modifications [17, 18]. Studies of viral gene expression in B cells infected in vitro show that upon infection, the W promoter (Wp) drives expression of EBNA-LP and EBNA2. As latency is established, the Wp becomes methylated and is largely silenced [19]. In some infections, the C promoter (Cp) is activated by EBNA2 and drives expression of all six EBNA genes. This pattern of latent gene expression is referred to as latency III [20]. In tumors and tumor-derived cell lines, two other patterns of latent gene expression are also recognized: latency I and II. These latency patterns differ in the expression of latency membrane proteins but share expression of EBNA1, the only EBNA family protein expressed in these latencies. In latency I and II, EBNA1 expression is driven by Q promoter (Qp), which is consistently unmethylated [21, 22]. In studies reported to date, there is consistent methylation of Cp in tumors, except in EBV-associated lymphoproliferative diseases occurring in profoundly immunosuppressed individuals [23] and some rare cases of EBV(+) peripheral T cell lymphoma [24]. Specific latency patterns have been recognized in association with particular tumor types: Burkitt lymphoma and gastric carcinoma show Latency I, and NPC and cHL show Latency II [8]. Methylation of the EBV Cp is also detectable in peripheral blood mononuclear cells from healthy individuals [25].

Given the importance of viral DNA methylation and its potential for recognizing different latency patterns and disease states, we developed a *m*ethylation-*s*pecific *P*CR for the *C P*romoter (MSPCP) to facilitate investigations of EBV DNA in clinical specimens. Our investigations using MSPCP include cell lines, formalin-fixed paraffin-embedded tissue specimens, plasma, and saliva.

## Materials and methods

### Cell culture and DNA isolation

Akata, B95.8, LCL, Namalwa, Rael, and Raji cells from laboratory stocks were cultured in complete RPMI media (RPMI, 10% FBS, 1% penicillin-streptomycin, 1% L-glutamine). BX1 Akata cells were grown in complete RPMI media supplemented with 1% geneticin (GibCo, Waltham, MA, #10131027). Cultures were maintained in 37 °C incubators with 5% CO_2_.

Lytic induction of Akata and BX1 Akata cells was done by culturing the cells in complete RPMI supplemented with anti-human IgG (MilliporeSigma, Burlington, MA, #I5260-1ML) at a concentration of 10 µg/mL for four days. Lytic induction of AG876, B95.8, Rael, and SNU-719 cells was done by culturing cells in complete RPMI supplemented with TPA at 20 ng/mL and sodium butyrate at 3 mM for four days. Supernatant media was filtered through a 0.45 μm filter (MilliporeSigma, Burlington, MA, #SLHVR33RS) and concentrated to 250 µL using Amicon Ultra-15 Centrifugal Filter Unit tubes (MilliporeSigma, Burlington, MA, #UFC910024). Concentrated virion media was treated with DNase I (New England Biolabs, Ipswich, Massachusetts, #M0303S) for 10 min at 37 °C to remove contaminating cellular DNA followed by the addition of 5mM EDTA. Cell and virion DNA was extracted with the DNeasy Blood and Tissue Kit (Qiagen, Germantown, MD, #69506) per manufacturer instructions and eluted in EB buffer (Qiagen, Germantown, MD, #19086). DNA concentrations were determined by Qubit Fluorometric Quantitation (Thermo Fisher Scientific, Waltham, MA, #Q32854).

### Human biological sample selection and DNA isolation

Tissue EBV status was determined by EBER in situ hybridization (EBER 1 DNP Probe, Roche Diagnostics, Indianapolis, IN, #760–1209). DNA was isolated from formalin-fixed paraffin-embedded (FFPE) tissue samples as previously described [26]. Briefly, FFPE sections were dissected from unstained slides. The tissue then underwent deparaffinization and DNA extraction using the automated Siemens Tissue Preparation System (Siemens Healthineers, Malvern, PA). Eluted DNA was stored at -20 °C. DNA concentrations were determined by Qubit, as above.

Peripheral blood specimens were collected at baseline (pretreatment) from people living with HIV with a diagnosis of classical Hodgkin lymphoma enrolled in AMC085 [27]. Samples were collected in EDTA or citrate anticoagulant collection tubes. Plasma was separated from whole blood by centrifugation and DNA was isolated from 500 µL of plasma using the QIAamp DNA blood mini kit (Qiagen, Germantown, MD, #51104) per manufacturer protocol.

Saliva samples were collected by the Johns Hopkins Stand-alone COVID Lab from students and staff as a part of universal screening during the COVID-19 pandemic. Approximately 5 mL of saliva was collected in 50 mL Falcon tubes containing no preservatives. Whole saliva was processed for total nucleic acid (TNA) using the MagMAX™ Viral/Pathogen II kit (Thermo Fisher Scientific, Waltham, MA, #A48383) on the same day as collection (< 12 h.). TNA was eluted at a volume of 50µL. Following SARS-CoV-2 testing, the remaining TNA (approximately 40 µL) was stored at -80 °C until further analysis. Only SARS-CoV-2-negative samples were used in this study.

EBV copy number was quantified by qPCR with a Namalwa cell DNA standard curve using TaqMan Universal Master Mix II (Thermo Fisher Scientific, Waltham, MA, #4440048), and custom primers and probes targeting the EBV BamW region [28].

### MSPCP primer design and synthetic templates

**Fig. 1:**
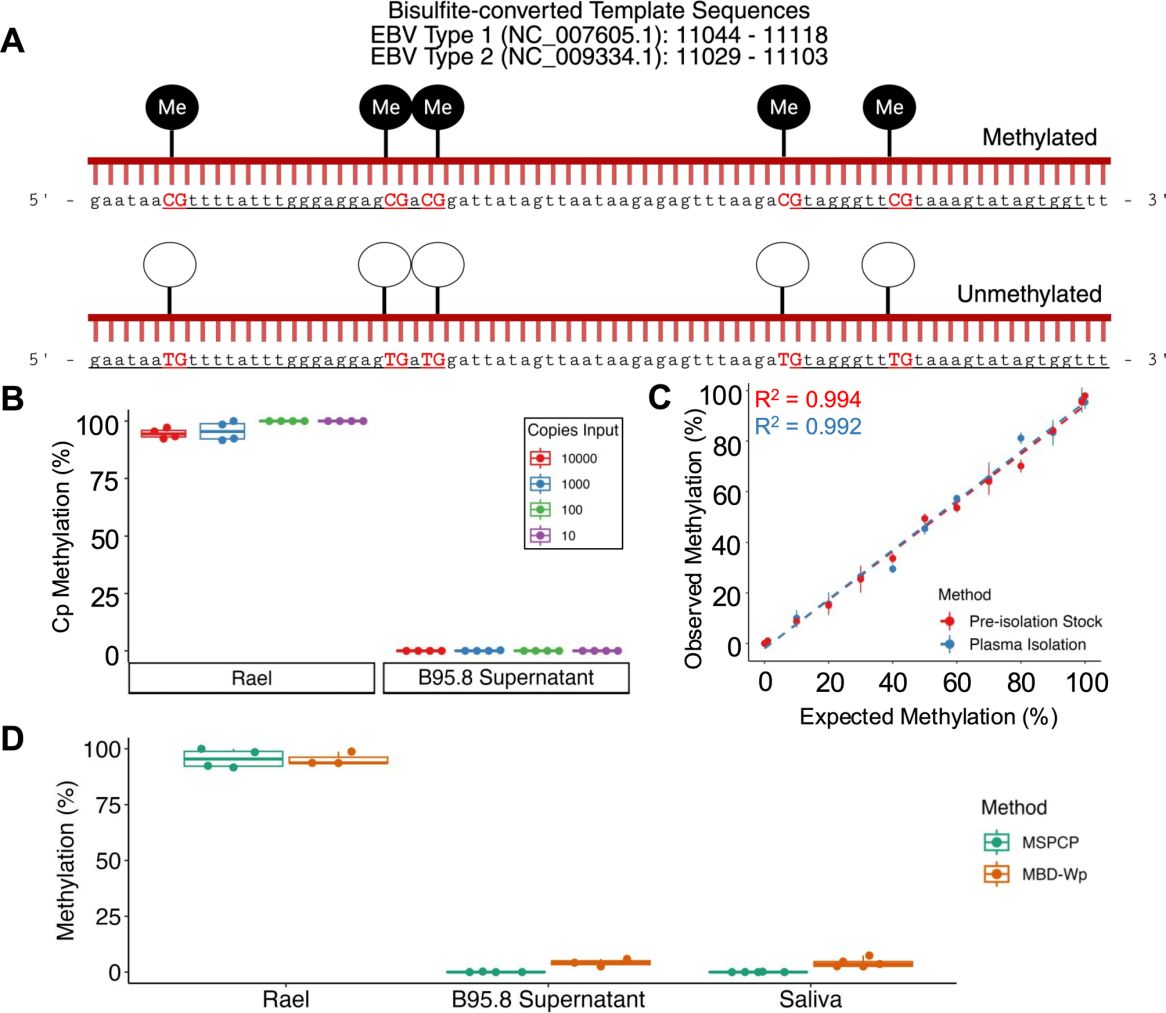
MSPCP quantifies EBV DNA methylation in low copy and heterogeneous samples. (**A**) Design schematic, target sequence, and coordinates for MSPCP. The top depicts the bisulfite-converted target sequence with methylated CpG sites. Below is the same sequence with unmethylated CpG sites. Primer binding sites are underlined. The U primer sets are elongated to coordinate M and U primer Tm. (**B**) Methylation in serial dilutions of 170 bp methylated (Rael) and unmethylated (B95.8 supernatant) DNA was quantified by MSPCP. (**C**) Rael cell DNA and B95.8 supernatant were mixed at known ratios and percent methylation was quantified to determine performance in heterogeneous mixtures (red; R^2^ = 0.994, Pearson correlation). These mixtures were spiked into normal human plasma, re-isolated, and quantified by MSPCP to determine if matrix effects affect quantification (blue; R^2^ = 0.992, Pearson correlation). (**D**) MSPCP (green) was compared to MethylMiner MBD quantification of methylation at the EBV Wp (orange) loci in Rael cell line DNA, B95.8 virion DNA, and EBV(+) saliva samples (*n* = 5). qPCR experiments were performed on 1,000 copies of EBV DNA per reaction and in technical duplicate

MSPCP primers were designed to target five CpG sites in the C promoter (Fig. 1A). Bisulfite-specific primers for Cp were designed with Primer Suite [29] using the EBV Type 1 genome (NC_007605.1), though selected primer sequences needed to show complete sequence homology to EBV Type 2 (NC_009334.1). Tm prediction of candidate primers was carried out using the OligoAnalyzer tool (Integrated DNA Technologies, Coralville, IA). Potential primer dimer identification was done using both IDT OligoAnalyzer and PrimerDimer [29]. Candidate primers with a dimerization ΔG of < -9.00 kcal/mole were excluded. An optimal primer annealing temperature was determined using a PCR temperature gradient ranging from 55 °C to 65 °C that was performed in technical duplicate.

Synthetic MSPCP template sequences were generated using bases 11,000–11,150 from the EBV Type 1 genome (NC_007605.1). Non-CpG C sequences in the primer binding site were changed to Ts corresponding with bisulfite-converted sequences. Cs occurring in CpG sites in the primer binding site were changed to T sequences in U-designated oligonucleotides and remained Cs in the M-designated oligonucleotides. Synthetic MSPCP templates were generated as gBlock Gene Fragments (Integrated DNA Technologies, Coralville, IA). As there are three CpGs in the forward primer binding site, and two CpGs in the reverse primer binding site, oligonucleotides were named MMM-MM or UUU-UU to represent the mimicked methylation status of each CpG. Oligonucleotides were resuspended per manufacturer recommendations and the concentration was confirmed using Tapestation High Sensitivity D1000 Tape and Reagents (Agilent, Santa Clara, CA, #5067–5584 and 5067–5585). All primer and oligonucleotide sequences can be found in Supplemental Table 1.

### Quantitative methylation analysis

Bisulfite conversion was carried out using the EZ DNA Methylation Lightning Kit (Zymo, Irvine, CA, #D5031) according to manufacturer instructions. Briefly, up to 20 µL of template DNA was mixed with 130 µL of Conversion Reagent. Samples were incubated in a thermocycler as follows: 95 °C for 15 min, 55 °C for 1 h, and 4 °C hold. Conversion was completed using the provided column and desulfonation reagents that also purified the DNA. Converted DNA was eluted at 21 µL in EB Buffer (Qiagen, Germantown, MD, #19086).

qPCR was performed using Power SYBR™ Green PCR Master Mix (Thermo Fisher Scientific, Waltham, MA, #4367660) at a reaction volume of 25 µL. Primers at a final concentration of 250 nM were used in parallel for all samples, with each reaction containing 5 µL of converted template. The synthetic MSPCP templates described above were used as standards for copy number quantification. A fully methylated control (Rael cell line DNA), an unmethylated control (B95.8 virion DNA), and a no-template control were included in each experiment. All samples and controls were run in technical duplicates. Using a Bio-Rad CFX96 PCR Detection System (Bio-Rad, Hercules, California, #1845097), qPCR was performed with the following settings: 95 °C for 10 min; 40 cycles of 95 °C for 30 s, 60 °C for 60 s. Melt curve analyses were performed using the instrument standard protocol.

### Mixed methylation and matrix effect analyses

DNA stocks of methylated and unmethylated EBV DNA were sheared to approximately 150 bp using a Covaris LE220-Plus Focused Ultrasonicator. Sheared stock mixtures were made by combining Rael cell line DNA and B95.8 virion DNA to achieve a final copy number of 10,000 copies/µL. To assess potential matrix effects of plasma on MSPCP, these mixed methylation stocks were spiked into normal human plasma (Innovative Research, Novi, MI, #IPLAWBK2E100ML), which had no prior detectable EBV DNA based on BamW qPCR as described above. Each sheared mixed methylation stock DNA was spiked into a plasma aliquot to achieve a final copy number of 20,000 copies/mL. Cell-free DNA was isolated from 500 µL of plasma containing spiked in EBV DNA and from normal plasma using the Qiagen Blood DNA Blood mini kit according to the manufacturer protocol.

### Orthogonal confirmation by bisulfite Sanger sequencing and MethylMiner MBD

To confirm MSPCP results, regions of interest of the EBV genome were amplified using native-sequence and bisulfite-sequence Cp M13-tagged primers described in Table S1. PCR amplicons were purified with the Qiagen PCR Purification Kit (Qiagen, Germantown, MD, #28106) according to manufacturer protocol. Single amplicon purity was confirmed with 1% agarose gel electrophoresis, visualized with GelGreen (Biotium, Fremont, CA, #41005). Amplified DNA was submitted to the Johns Hopkins University Genetic Resources Core Facility for Sanger sequencing of forward and reverse reads. Sequencing traces were analyzed using the Chromas software (Technelysium, South Brisbane, Australia).

The MethylMiner Methylated DNA Enrichment assay (Thermo Fisher Scientific, Waltham, MA, #ME10025) was also performed as previously described [28]. Briefly, streptavidin Dynabeads were incubated with Methyl-CpG Binding Domain-biotin (MBD-biotin). Next, DNA was sonicated to 600 bp and spiked into K562 cell line carrier DNA (Promega, Madison, WI, #E4931) to bring the final loading amount to 1 µg of DNA. Samples were incubated with MBD-coupled beads, then supernatants were collected as no-capture DNA. DNA was then eluted sequentially in increasing concentrations of sodium elution buffer at 300 mM, 450 mM, and 2000 mM. EBV present in the no-capture DNA and all eluted fractions of DNA was quantified separately in technical duplicate with BamW qPCR as described above.

### Data analysis

All qPCR data were analyzed using CFX Maestro software (Bio-Rad). MSPCP percent methylation was calculated as a percentage of absolute copy number using the formula $$\:\varvec{P}\varvec{e}\varvec{r}\varvec{c}\varvec{e}\varvec{n}\varvec{t}\:\varvec{m}\varvec{e}\varvec{t}\varvec{h}\varvec{y}\varvec{l}\varvec{a}\varvec{t}\varvec{i}\varvec{o}\varvec{n}=$$$$\:\frac{\varvec{M}\:\varvec{C}\varvec{o}\varvec{p}\varvec{i}\varvec{e}\varvec{s}}{\varvec{M}\:\varvec{C}\varvec{o}\varvec{p}\varvec{i}\varvec{e}\varvec{s}+\varvec{U}\:\varvec{C}\varvec{o}\varvec{p}\varvec{i}\varvec{e}\varvec{s}}$$ $$\:\times\:\:100$$. Percent methylation by the MethylMiner assay was calculated as $$\:\varvec{P}\varvec{e}\varvec{r}\varvec{c}\varvec{e}\varvec{n}\varvec{t}\:\varvec{m}\varvec{e}\varvec{t}\varvec{h}\varvec{y}\varvec{l}\varvec{a}\varvec{t}\varvec{i}\varvec{o}\varvec{n}=$$$$\:\frac{350\mathbf{m}\mathbf{M}+400\mathbf{m}\mathbf{M}+2000\mathbf{m}\mathbf{M}\:\mathbf{f}\mathbf{r}\mathbf{a}\mathbf{c}\mathbf{t}\mathbf{i}\mathbf{o}\mathbf{n}\:\mathbf{c}\mathbf{o}\mathbf{p}\mathbf{i}\mathbf{e}\mathbf{s}}{\mathbf{N}\mathbf{o}-\mathbf{c}\mathbf{a}\mathbf{p}\mathbf{t}\mathbf{u}\mathbf{r}\mathbf{e}+\:350\mathbf{m}\mathbf{M}+400\mathbf{m}\mathbf{M}+2000\mathbf{m}\mathbf{M}\:\mathbf{f}\mathbf{r}\mathbf{a}\mathbf{c}\mathbf{t}\mathbf{i}\mathbf{o}\mathbf{n}\:\mathbf{c}\mathbf{o}\mathbf{p}\mathbf{i}\mathbf{e}\mathbf{s}}$$ $$\:\times\:\:100$$. Data analysis, plotting and statistical comparisons were performed using RStudio (version 2024.09.1+394). Correlation coefficients were determined by Pearson correlation test.    

## Results

### MSPCP detects Cp methylation in tumor cell lines but not in lymphoblastoid cell lines or virion DNA

The efficacy of MSPCP was first assessed in DNA extracted from virion supernatants, lymphoblastoid cell lines, EBV(+) Burkitt lymphoma cell lines (Rael and AG876), an NPC cell line (C666-1), and an EBV(+) gastric carcinoma cell line (SNU-719) (Table 1A). As expected, virion supernatants did not show methylation at Cp. Similarly, lymphoblastoid cell lines did not show methylation at Cp. The Burkitt lymphoma, NPC, and gastric carcinoma cell lines, however, showed high level methylation at Cp, except for the AG876 cell line. AG876 is among the Burkitt cell lines that have drifted from latency I as evidenced by expression of EBNA2 and the Cp has previously been reported to be unmethylated [30]. Bisulfite Sanger sequencing provided orthogonal confirmation of the MSPCP results (Supplemental Figure S1).

**Table 1:** MSPCP quantifies methylation in EBV(+) cell lines and FFPE tumor samples

**A. Cell Lines and Synthetic Templates**		**Mean C Promoter Methylation (s.d.)**
Synthetic Oligos	Methylated Oligo Template	99.98% (< 0.01)
	Unmethylated Oligo Template	< 0.01% (0.01)
Latency I	C666-1	97.38% (0.25)
	Rael	96.99% (1.33)
	SNU-719	88.27% (0.61)
Latency III	AG876	0.69% (0.11)
	B95.8	< 0.01% (< 0.01)
	LCL	< 0.01% (< 0.01)
Induced Virion Supernatant	AG876 Virus	0.39% (0.03)
	Akata Virus	5.96% (1.87)
	BX1 Akata Virus	1.87% (0.11)
	B95.8 Virus	< 0.01% (< 0.01)
	SNU-719 Virus	1.57% (0.29)
**B. EBV(+) Tissue Samples**		**C Promoter Methylation**
Lymphomas	Classical Hodgkin Lymphoma; HIV(+)	100.00%
	Classical Hodgkin Lymphoma; HIV(+)	99.83%
	Classical Hodgkin Lymphoma; HIV(+)	98.80%
	Classical Hodgkin Lymphoma; HIV(+)	97.30%
	Classical Hodgkin Lymphoma; HIV(+)	94.19%
	Diffuse Large B-Cell Lymphoma	100.00%
	Extranodal NK/T Cell Lymphoma	100.00%
Carcinomas	Gastric Adenocarcinoma	100.00%
	Nasopharyngeal Carcinoma	99.90%
	Nasopharyngeal Carcinoma	99.88%
Non-cancer biopsy	Lymphoid Hyperplasia	3.40%

(**A**) MSPCP quantifies high Cp methylation in latency I cell lines, and minimal Cp methylation in latency III cell lines and virion-containing supernatant media. (**B**) MSPCP quantifies high Cp methylation in all ten EBV(+) tumor types analyzed. The method also quantified minimal Cp methylation in a non-cancerous EBV(+) tissue biopsy

Next, we analyzed the lowest EBV copy number input to the reaction in which MSPCP could quantify Cp methylation using serial dilutions of Rael cell or B95.8 virion DNA (Fig. 1B). We found that results were reproducibly methylated or unmethylated across serial dilutions ranging from 10,000 initial EBV copies down to 10 initial EBV copies.

In order to assess accuracy of methylation measurements by MSPCP, we analyzed mixtures of methylated and unmethylated EBV DNA each totaling 10,000 copies (Fig. 1C). Methylation percentages observed with MSPCP correlated closely with the expected percent methylation (R^2^ = 0.994) down to 1% methylated DNA. To evaluate potential matrix effects from plasma [31], MSPCP was performed on the same mixture of methylated EBV DNA re-isolated from plasma containing the DNA mixtures as spike-ins. Similar to the stock methylated mixtures, MSPCP accurately measured each methylated fraction down to 1% with a correlation coefficient of R^2^ = 0.992.

Next, we compared MSPCP to the MBD-bead methylation, which utilizes primers targeting the BamW region [28]. DNA extracted from the Rael cell line and B95.8 supernatant (virion) DNA were analyzed. Both methods yielded concordant results at 1,000 copies of EBV (Fig. 1D). We have observed that, compared to MBD, the MSPCP method provides more reproducible results in low copy number samples, as low as 10 copies of starting material, when the two methods are compared directly in cell line DNA (data not shown). Saliva specimens (*n* = 5) were tested by both methods with 1,000 EBV copies input in each reaction. MSPCP and MBD-bead capture followed by BamW quantification showed virtually no methylation, as expected.

To refine the procedure, we tried to find a potential stopping point, after bisulfite conversion prior to MSPCP, by storing the converted DNA at predetermined conditions before proceeding to qPCR (Supplemental Figure S2). Rael and B95.8 supernatant were mixed at known percentages and these samples were stored in specified conditions prior to MSPCP quantification. We found that performing nonstop analysis is preferred, though storage at 4 °C overnight is a minimally disruptive alternative.

### MSPCP quantifies EBV DNA methylation in human biological samples

**Fig. 2:**
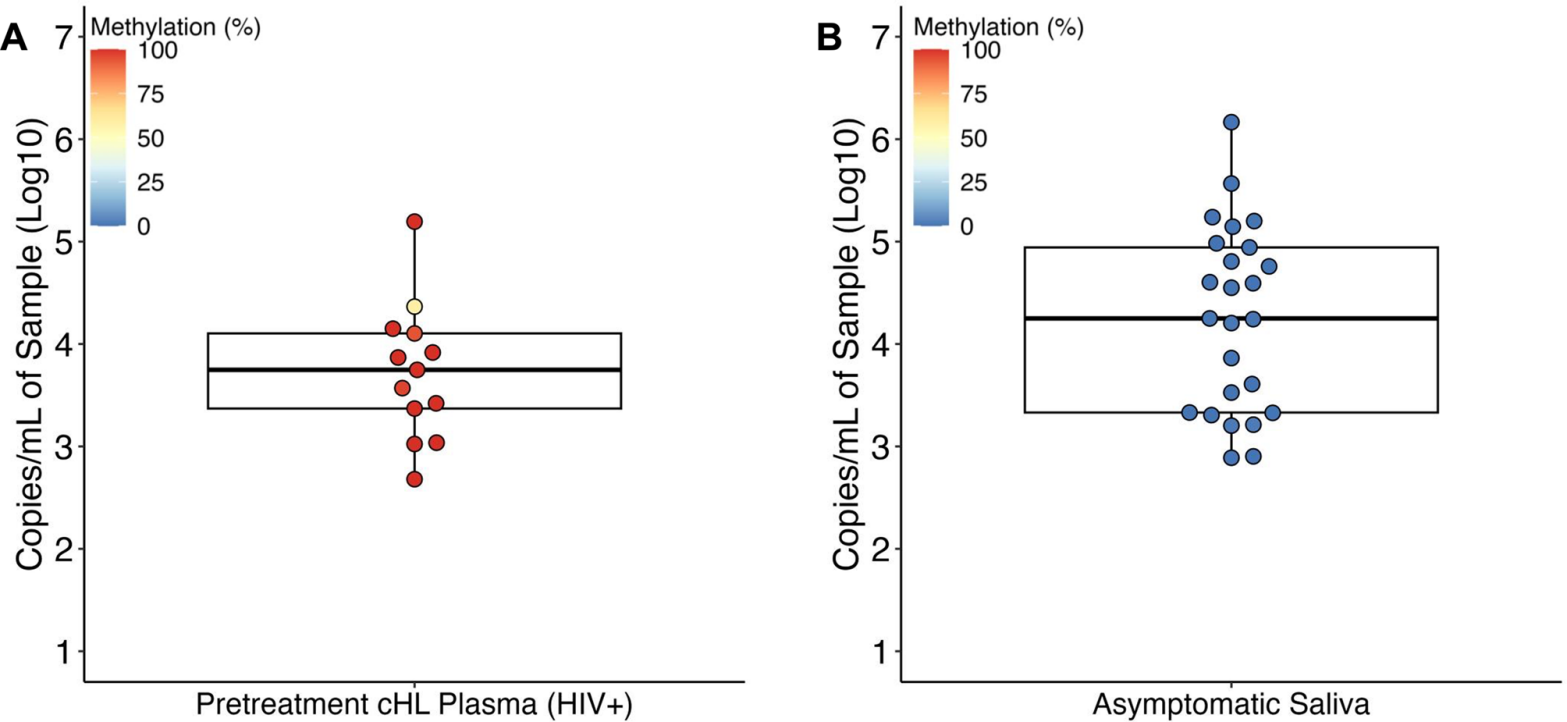
MSPCP quantifies EBV DNA methylation in plasma and saliva isolates. (**A**) EBV copy number in plasma from pretreatment Hodgkin lymphoma patients (*n* = 13) ranges from 2.68 to 5.19 log_10_ copies per mL of plasma (median: 3.75). One sample is moderately methylated (58.22%), and all others are densely methylated at Cp (range: 94.33–100%). (**B**) EBV copy number in saliva of non-selected general university staff and students (*n* = 25) ranges from 2.88 to 6.16 log_10_ copies per mL of saliva (median: 4.25). These samples have little to no Cp methylation (range: 0–1.75%)

We applied MSPCP to ten FFPE tissue biopsies from a variety of EBV(+) cancers and one case of EBV(+) lymphoid hyperplasia (Table 1B). Median input copy number for MSPCP was 4,890 EBV copies (range: 430–160,846). Each of the tumor samples showed high level methylation (median = 99.8%, range = 94–100%), while the lymphoid hyperplasia sample showed minimal methylation (3%). Next, we examined Cp methylation in plasma EBV DNA from 13 Hodgkin lymphoma patients with HIV (Fig. 2A) and found that the EBV DNA was almost entirely methylated at Cp (median = 100%, range = 58.22–100%). In situ hybridization of the cHL tumor from the patient with 58.22% Cp methylation confirmed EBV EBER RNA was present in the tumor’s Reed-Sternberg cells. Analysis of saliva from 25 university adults with detectable saliva EBV DNA (Fig. 2B) showed that Cp was essentially unmethylated in all of these samples (median = 0%, range = 0–1.75%).

## Discussion

This study describes the design, validation, and application of MSPCP to evaluate EBV DNA methylation at the Cp locus in a variety of contrived and clinical human biological specimens. MSPCP shows high accuracy and linearity in contrived samples, and these results are not affected by plasma matrix effects. As expected, Cp methylation was detected in EBV-associated tumors and in plasma from patients with EBV(+) Hodgkin lymphoma. In contrast, Cp methylation was not detected, or detected at only at very low level, in EBV-associated lymphoid hyperplasia and in saliva from an unselected university population.

Cp was chosen as the target locus due to prior literature demonstrating that this region is consistently methylated in EBV(+) cancers, but less methylation is present in non-malignant EBV-associated diseases, such as infectious mononucleosis [32, 33]. Additionally, Cp differs from Wp as a non-repetitive region, while EBV genomes contain approximately 8 repeats of the Wp cassette [34, 35]. These cassettes may be differentially methylated in each repeat, potentially making data interpretation more complex. MSPCP was designed, in part, to be accessible and straightforward to interpret. Targeting Wp may offer higher sensitivity due to the increased copy number available per genome, but differential melt temperature analysis may be a preferrable method of analysis, given the number of CpG sites involved [5, 36, 37].

EBV virions are never methylated, even when they derive from tumor cells undergoing lytic replication. This is because the virion DNA is synthesized by the viral DNA polymerase subunit, BALF5, rather than cellular DNA polymerases [16, 38]. The virion DNA polymerase does not interact with the cellular DNA methyltransferase to perpetuate DNA methylation following synthesis of new DNA [39]. This natural process makes EBV DNA methylation an attractive method to differentiate circulating lytic virion DNA from latent cell/tumor-derived DNA molecules. Low level methylation seen in some virion supernatants (Table 1B) is likely contaminating genomic DNA from lysed cells. This is most observable in BL cell lines, which are Latency I cell lines with dense methylation at latency promoters, including Cp.

We demonstrate that MSPCP is effective in FFPE tissue samples, which enables studies of archival samples. FFPE samples are known to have issues with DNA integrity, such as DNA-protein crosslinking that can impact nucleic acid integrity and introduce artifacts [40, 41]. A previous study demonstrated that crosslinking or spontaneous deamination in FFPE-derived DNA does not negatively impact methylation calling compared to frozen samples [42]. Likewise, MSPCP does not underperform in FFPE versus frozen samples despite crosslinking and can be applied to archival samples effectively.

In populations at high risk for EBV(+) malignancy, such as southern Chinese populations at risk for NPC, detection of EBV DNA in plasma has shown promise for early cancer detection [10, 18, 43]. In their initial study, these investigators screened plasma from 20,174 men and if EBV DNA was detected repeating the screen at approximately 4 weeks. In the initial screen, EBV DNA was detected in 5.5% of participants. At follow-up 27.8% of these individuals were persistently positive. Among persistently positive participants, 34 (11%) were found to have NPC. In follow up reports, these investigators used bisulfite sequencing to characterize patterns of methylation of the viral DNA, or fragmentomic investigations to infer patterns of viral DNA methylation, increased the specificity of EBV DNA detection for the diagnosis of NPC [18, 33]. These investigations relied upon deep sequencing.

EBV infection spreads through salivary contact and salivary EBV can be used to immortalize cord blood B cells [44]. Since virion DNA is not methylated [16], our finding that EBV in the saliva from healthy adults is unmethylated was expected. The present finding that CpG methylation at Cp is almost entirely absent from viral DNA in saliva is consistent with the hypothesis that salivary EBV DNA in healthy individuals consists of largely of virion DNA. Given this, a group in Guangzhou investigated EBV methylation in saliva, oropharyngeal swabs, oral swabs and mouthwash [45, 46]. These investigators reported that assay for methylated viral DNA increased the specificity for NPC over EBV copy number alone.

In an interesting report from Kenya, blood and saliva specimens were studied from children with and without malaria [47]. High EBV DNA copy numbers were detected in plasma and saliva irrespective of malaria, and copy number in plasma decreased in children without malaria. Methylation was assessed at three loci in the viral genome by a technique that involved bisulfite treatment, PCR amplification, and matrix-assisted laser desorption ionization time-of-flight (MALDI-TOF) mass spectrometry for quantitative methylation detection. The investigators reported that EBV methylation was lower in the malaria group in plasma and saliva and inferred that malaria was associated with increased lytic viral replication.

MSPCP has some limitations and the interpretation of the findings has some caveats. A limitation is that deletions of the EBV Cp locus have been reported in some cancers [48]. A caveat to keep in mind with regard to interpretation is that while we have presented evidence that methylated EBV C promoter DNA is found in the plasma of patients with active EBV(+) tumors [25], application of the assay to whole blood would almost certainly find methylated Cp DNA insofar as the region is methylated in the lymphocytes of healthy individuals. Because in healthy individuals, the pool of latently infected lymphocytes is almost exclusively in resting memory B cells [49], which are a very minor contributor to plasma DNA, this “normal” methylation is not expected to be commonly detected in plasma. Similarly, some patients with EBV(+) or EBV(-) cancers have high EBV copy numbers in their plasma that is likely of virion origin, particularly in patients with HIV [50, 51]. We believe this may be the cause of the 58% methylated cHL plasma sample included in this study (Fig. 2A), though further testing is required to support this hypothesis. Lastly, Cp is known to be methylated in Latency I and II, but unmethylated in Latency III [8]. MSPCP may be less relevant for the study of Latency III-associated diseases, such as some of the post-transplant lymphoproliferative disorders [20].

Interest in EBV DNA as a biomarker for diagnosis and monitoring of a variety of cancers continues to grow. As noted above, very sophisticated methods to better characterize EBV DNA, particularly in plasma so as to increase the specificity for malignancy, are being explored. Bisulfite deep sequencing, fragmentomics and mass spectrometry have all been investigated with promising results. Here we describe a simple PCR-based assay. In a previous investigation, we explored the use of magnetic beads coupled with methyl-CpG binding domain protein to enrich methylated DNA from plasma cell-free DNA, followed by PCR to assess the EBV DNA in the fraction that bound to the beads versus the flow through [28]. That approach offered a general assessment of CpG methylation in a region whereas the present approach is focused on specific CpG sites. Nonetheless, we showed that MSPCP provides concordant data compared to this existing assay, but offers more specificity with regard to the particular sites of methylation, faster experiment turnaround time, and a lower EBV DNA input requirement.

## Conclusions

We demonstrate that MSPCP can quantify Cp methylated EBV DNA in biopsy specimens, plasma, and saliva. Access to sophisticated deep sequencing or mass spectrometry equipment is not necessary and the assay is straightforward and fast. We are presently investigating the application of the assay for facilitating cancer diagnoses in a variety of high-risk populations.

### Patents

A provisional patent application, serial number 63/714,692, for “Early Diagnosis of Cancer Through EBV Promoter Methylation” has been filed with the United States Patent and Trademark Office.

## Supplementary Information

Below is the link to the electronic supplementary material.

**Supplementary Material 1:** MSPCP published data

**Supplementary Material 2: Supplemental Figure S1**. Bisulfite Sanger sequencing of MSPCP target site. Rael (top), a Latency I EBV(+) Burkitt lymphoma cell line, has methylated CpG sites at the MSPCP target sequence. B95.8, a Latency III LCL cell line, has unmethylated CpG sites at the same loci. These results are concordant with MSPCP quantification in Table 1A. **Supplemental Figure S2**. Analysis of stop points during MSPCP assay. To determine samples can be placed on hold following bisulfite conversion, reproducibility across different storage conditions was analyzed (top). Immediate PCR = samples were analyzed by MSPCP qPCR immediately after conversion. 2hr Delay = samples were kept at RT for two hours, to simulate performing sequential batches of MSPCP qPCRs. 4°C Overnight and − 20°C Overnight = samples were stored at 4°C or -20°C overnight, respectively, before MSPCP analysis. Rael and B95.8 supernatant DNA were mixed at known percentages and observed percent methylation was quantified by MSPCP. Each reaction was performed in triplicate. Bars represent standard deviation of replicates. R^2^ was determined by Pearson correlation. m refers to the slope of the fit line in each condition. **Supplemental Table 1**. Details of PCR reagents used for described experiments

## Data Availability

The original data presented in the study are included in the supplemental materials. Any other original data will be available on request from the corresponding author.

## Abbreviations

EBV: Epstein-Barr Virus
Cp: C Promoter
Wp: W Promoter
MSPCP: Methylation-specific PCR for the EBV C Promoter
PCR: Polymerase Chain Reaction
qPCR: Quantitative Polymerase Chain Reaction
FFPE: Formalin-fixed Paraffin-embedded
MBD: Methyl-CpG Binding Domain
DLBCL: Diffuse Large B Cell Lymphoma
cHL: Classical Hodgkin Lymphoma
HIV: Human Immunodeficiency Virus
NPC: Nasopharyngeal Carcinoma
NK/T Cell Lymphoma: Natural Killer/T Cell Lymphoma
LCL: Lymphoblastoid Cell Line
