# Supplementary material for: Methylation-specific qPCR for the EBV C promoter to quantify EBV methylation
Source: Infect Agent Cancer. 2025 Oct 30;20:75. doi: 10.1186/s13027-025-00702-x (PMC12574023; doi:10.1186/s13027-025-00702-x)
Supplement: Supplementary file 2 — Supplementary Material 2: Supplemental Figure S1. Bisulfite Sanger sequencing of MSPCP target site. Rael (top), a Latency I EBV(+) Burkitt lymphoma cell line, has methylated CpG sites at the MSPCP target sequence. B95.8, a Latency III LCL cell line, has unmethylated CpG sites at the same loci. These results are concordant with MSPCP quantification in Table 1A. Supplemental Figure S2. Analysis of stop points during MSPCP assay. To determine samples can be placed on hold following bisulfite conversion, reproducibility across different storage conditions was analyzed (top). Immediate PCR = samples were analyzed by MSPCP qPCR immediately after conversion. 2hr Delay = samples were kept at RT for two hours, to simulate performing sequential batches of MSPCP qPCRs. 4°C Overnight and − 20°C Overnight = samples were stored at 4°C or -20°C overnight, respectively, before MSPCP analysis. Rael and B95.8 supernatant DNA were mixed at known percentages and observed percent methylation was quantified by MSPCP. Each reaction was performed in triplicate. Bars represent standard deviation of replicates. R2 was determined by Pearson correlation. m refers to the slope of the fit line in each condition. Supplemental Table 1. Details of PCR reagents used for described experiments [file 13027_2025_702_MOESM2_ESM.docx]

**Supplemental Figure S1**: **Bisulfite Sanger sequencing of MSPCP target site**. Rael (top), a Latency I EBV(+) Burkitt lymphoma cell line, has methylated CpG sites at the MSPCP target sequence. B95.8, a Latency III LCL cell line, has unmethylated CpG sites at the same loci. These results are concordant with MSPCP quantification in Table 1A.

**Supplemental Figure S2**: **Analysis of stop points during MSPCP assay**. To determine samples can be placed on hold following bisulfite conversion, reproducibility across different storage conditions was analyzed (top). Immediate PCR = samples were analyzed by MSPCP qPCR immediately after conversion. 2hr Delay = samples were kept at RT for two hours, to simulate performing sequential batches of MSPCP qPCRs. 4°C Overnight and − 20°C Overnight = samples were stored at 4°C or -20°C overnight, respectively, before MSPCP analysis. Rael and B95.8 supernatant DNA were mixed at known percentages and observed percent methylation was quantified by MSPCP. Each reaction was performed in triplicate. Bars represent standard deviation of replicates. R^2^ was determined by Pearson correlation. m refers to the slope of the fit line in each condition.

**Supplemental Table 1**: **Details of PCR reagents used for described experiments**.

**Supplemental Figure S1**


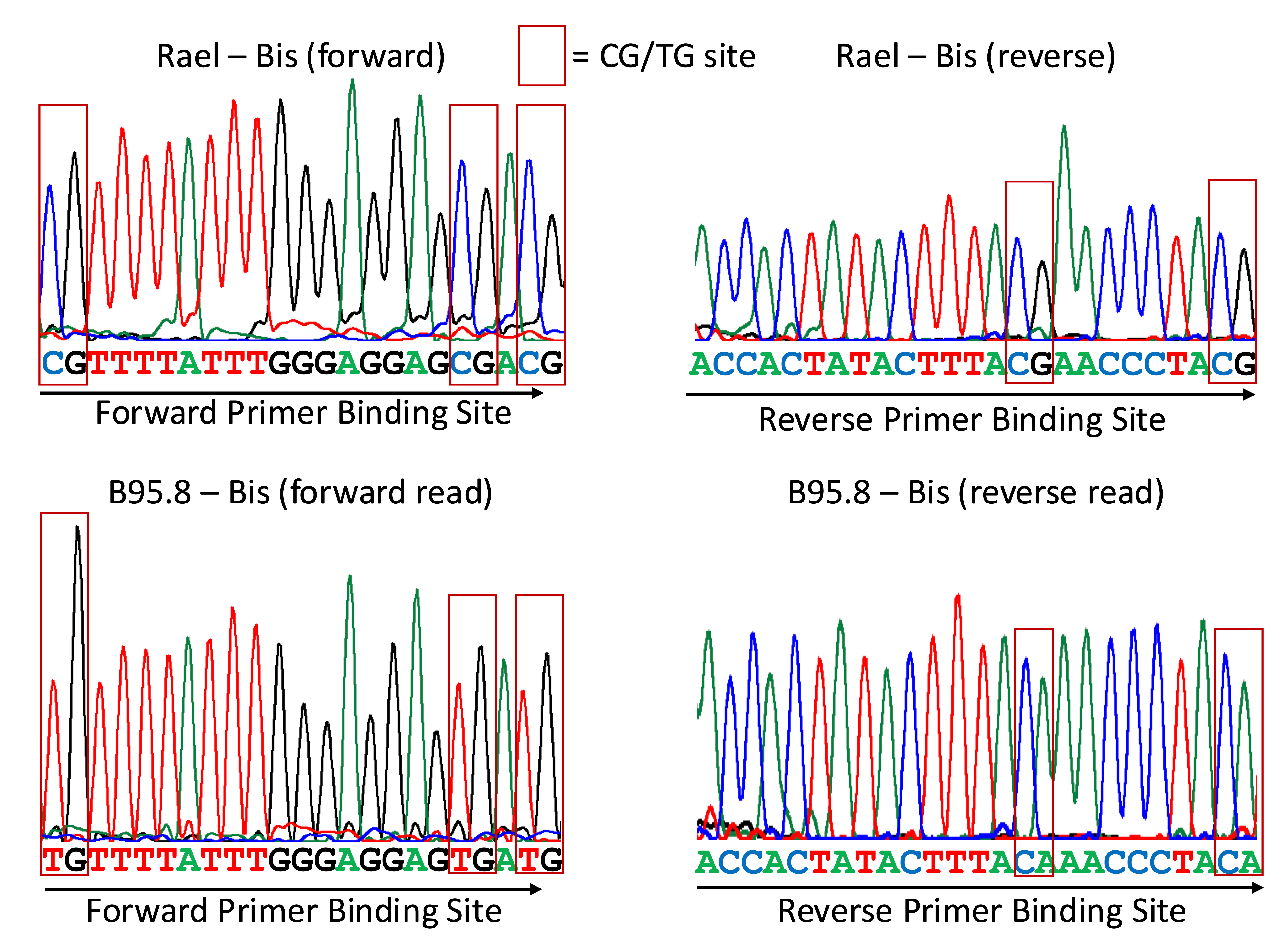


**Supplemental Figure S2**

**
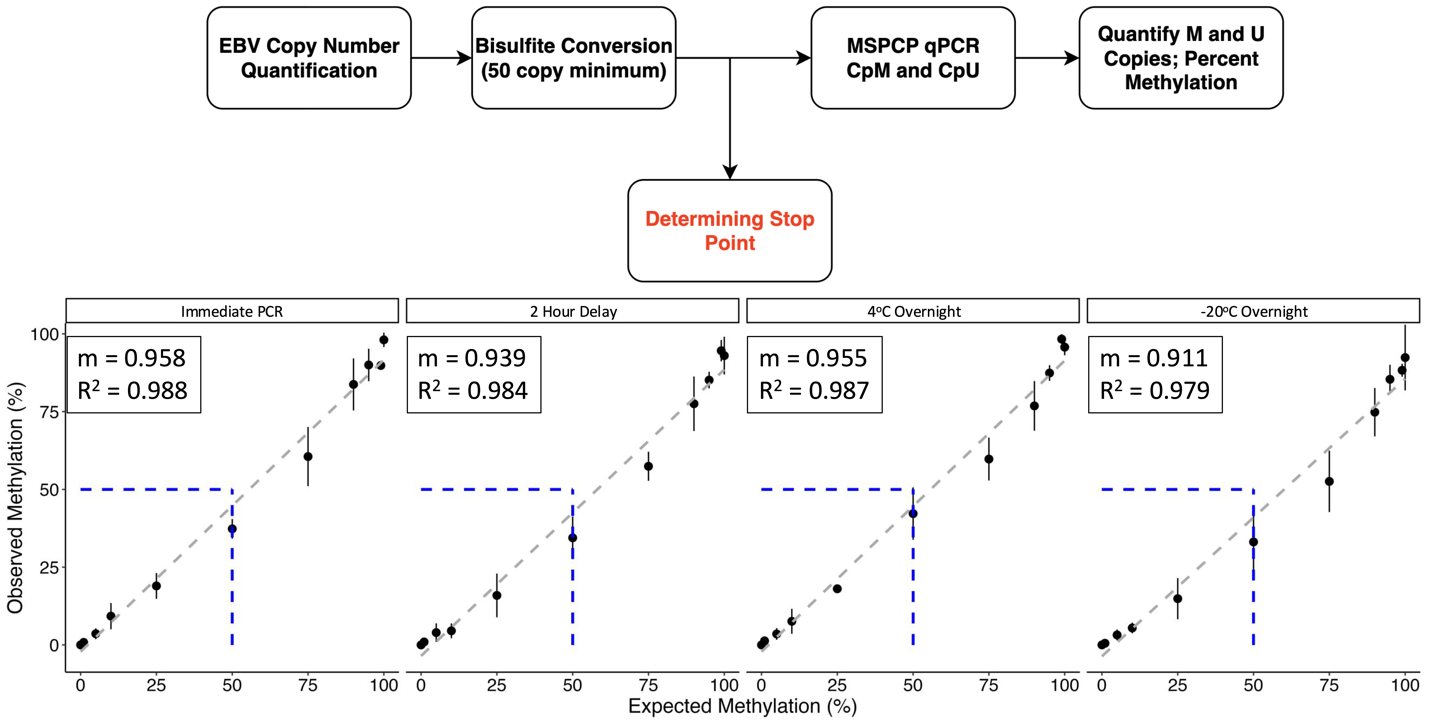
**

**Supplemental Table 1**

| **Primer Name** | **Forward Sequence** | **Reverse Sequence** | **Probe Sequence** | **Notes** |
| --- | --- | --- | --- | --- |
| BamW_Native | CCCAACACTCCACACC | TCTTAGGAGCTGTCCGAGGG | 5'-/56-FAM/CACACACTACACACACCCACCCGTCTC/3BHQ_1/-3’ | Primers described in Shamay et al., 2020. *Blood Advances*. |
| Cp_Native_M13 | TGTAAAACGACGGCCAGTGTTGAGAGGTTAGTGTTTTAAATATGTATTTTAGG | CAGGAAACAGCTATGACCTAAAACCCCTTTACCCAACCC |  | M13 tag is underlined. |
| Cp_Bisulfite_M13 | TGTAAAACGACGGCCAGTTGTTATAAGATTATTAAGTTGGTGTAAA | CAGGAAACAGCTATGACCTTTACAACAAAACACAAAATTTTTATAA |  | M13 tag is underlined. |
| MSPCP_M | **CG**TTTTATTTGGGAGGAG**CG**A**C** | ACCACTATACTTTA**CG**AACCCTA**CG** |  | Target CpG sites in bold. |
| MSPCP_U | GAATAA**TG**TTTTATTTGGGAGGAG**TG**A**T** | AAACCACTATACTTTA**CA**AACCCTA**CA** |  | Target CpG sites in bold.  U Primers are extended in underlined regions at 5’ end to match Tm of M primers |
| MSPCP_MMM-MM_Gblock | ACCTTGTTGGCGGGAGAAGGAATAA**CGTT**TTATTTGGGAGGAG**CG**A**CG**GATTATAGCCAATAAGAGAGCTCAAGA**CG**TAGGGTT**CG**TAAAGTATAGTGGTTTCGTGGGACCTTAGAGGTGGAGCA |  |  | Target CG sites in bold.  Synthetic template for MMM-MM. Primer sites are underlined. |
| MSPCP_UUU-UU_Gblock | ACCTTGTTGGCGGGAGAAGGAATAA**TG**TTTTATTTGGGAGGAG**TG**A**TG**GATTATAGCCAATAAGAGAGCTCAAGA**TG**TAGGGTT**TG**TAAAGTATAGTGGTTTCGTGGGACCTTAGAGGTGGAGCA |  |  | Target CG sites in bold.  Synthetic template for UUU-UU. Primer sites are underlined. |
